# Rs7537605 polymorphism in VAV3 gene and rs28665122 polymorphism in SEPS gene are not associated with Hashimoto's thyroiditis in North-East Algerian population

**DOI:** 10.4314/ahs.v22i4.30

**Published:** 2022-12

**Authors:** Warda Kherrour, Souheyla Benbia, Leila Hambaba, Dean Kaličanin

**Affiliations:** 1 Biotechnology's Laboratory of the Bioactive Molecules and the Cellular Physiopathology, Department of Biology of Organisms, Faculty of Natural and Life Sciences, University of Batna 2, Batna, Algeria; 2 Biotechnology's Laboratory of the Bioactive Molecules and the Cellular Physiopathology, Department of Microbiology and Biochemistry, Faculty of Natural and Life Sciences, University of Batna 2, Batna, Algeria; 3 Department of Medical Biology, University of Split School of Medicine, Split, Croatia

**Keywords:** Hashimoto's thyroiditis, inflammation, hypothyroidism, rs7537605, rs28665122, Algerian population

## Abstract

**Background:**

Hashimoto's thyroiditis (HT) is the most common form of autoimmune thyroid disease which leads, in most cases, to hypothyroidism. HT is also classified as a multifactorial disease, which is caused by an interaction between genetic and environmental factors. Current knowledge of HT genetics is still very limited, especially in Algerian population.

**Objective:**

We wanted to investigate the association of two single-nucleotide polymorphisms (SNPs) inside VAV3 and SEPS genes with HT in Algerian population.

**Methods:**

We conducted a case-control study that included 100 HT cases and 126 healthy controls that were recruited from three private endocrinology clinics. Two SNPs, rs7537605 and rs28665122 inside VAV3 and SEPS genes were genotyped using real-time polymerase chain reaction (real-time PCR). Binary logistic regression model was used to test the association of selected SNs with HT and linear regression model was used to test association of these SNPs with thyroid peroxidase antibodies (TPOAb) levels.

**Results:**

Binary logistic regression results revealed no allelic association of the minor allele A between Hashimoto's thyroiditis cases and healthy controls (P=0.896) for the rs7537606 in VAV3 gene. The same observation was reported for the AA (P=0.477), AG (P=0.752) genotypes and for the genotypic models: dominant (P=1.0) and recessive (P=0.555). Also, there was no significant difference in the TT (P=0.230), TC (P=0.717) and allelic distribution of the minor allele T (P=0.859), and the combined models: TT + TC (P=1.0), TC + CC (P=0.138) between patients and controls for the rs28665122 polymorphism of the SEPS1 gene.

**Conclusion:**

This is the first genetic study that investigated the genetic association of rs7537605 and rs28665122 inside VAV3 and SEPS genes in Algerian population. Our results suggest that these two SNPs may not be involved in the pathogeneses of HT since we found no association between them and HT/TPOAb levels. Further research that will include larger sample size is required.

## Introduction

Hashimoto's thyroiditis (HT) is the most common inflammatory autoimmune thyroid disorder (AITD) that leads to hypothyroidism in more than 20% of patients. It has different clinical appearance, from mild subclinical hypothyroidism with thyroid-stimulating hormone (TSH) level just slightly above the normal reference range and with normal level of free thyroxine (fT4), to overt hypothyroidism with low serum of fT4 and elevated level of TSH, especially in iodine-sufficient areas^1^,^2^. Various inflammatory markers have been associated with the disease including elevated red cell distribution width (RDW) values^3^, increased neutrophil-to-lymphocyte ratio (NLR) 4 and uric acid to HDL cholesterol ratio (UHR) 5.

Several epidemiological studies have shown that prevalence of HT is increased with age, and it occurs in women more frequently than in men and depends on the geographic location 6.

The diagnosis of HT is based on well-defined clinical criteria, with thyroid ultrasound, presence of a firm and irregular goitre and by the presence of very high levels of autoantibodies against thyroid peroxidase (TPOAb) 7. In the case of the absence of TPOAb, the presence of antibodies against thyroid thyroglobulin (TgAb) can confirm the diagnosis 8.

Danish population-based twin genetic studies have shown an increased prevalence of AITD in monozygotic twins (29% to 55%) compared to dizygotic twins (0–7%) 7. Results of the above-mentioned studies confirmed that approximately 75% of AITD phenotypic variance is due to genetic factors and the rest is due to environmental factors which make HT a multifactorial disease 7.

To determine genetic susceptibility to HT, there were performed a large number of different studies but genetic background is still limited to date. Case-control studies determined three genetic markers associated with HT: *HLA-DR, CTLA-4*, and *PTPN22* 9 - 12. Furthermore, other studies identified new genes that may be associated with HT, such as *Tg* 13, *VDR* 14, *SH2B*^3^, *PDE8B* 15, *IL*^15^ ^16^, *IL27* 17, *FOXP3* 18, and *IRAK1* 19.

One study targeted the Selenoprotein S (*SEPS1*) gene in the pathogens of HT. It is interesting because it is shown that selenium has great effect on the thyroid gland and autoimmunity 20 - 22. A significant association between the rs28665122 located on the *SEPS1* gene and HT has been reported; where the risk for reported thyroiditis is modulated by the interaction between the promoter of *NFE2L2* and *SEPS1* gene 21,22.

Furthermore, Oryoji et al.^23^ performed a genome-wide association analysis (GWAS) and replication study where they compare patients with HT and patients with Graves' disease (GD) and found that rs7537605 located at *VAV3* gene was significantly associated with HT-related hypothyroidism. A recent and first GWAS study performed in HT patients only identified three genetic SNPs associated with the disease: rs12944194 located 206 kb from *SDK2*, rs75201096 inside *GNA14*, and rs791903 inside *IP6K3* 24. In the present study, we aimed to test the association of two genetic single nucleotide polymorphisms (SNPs) from two previous studies 21,23 that are related to HT (rs7537605 inside *VAV3* gene and rs28665122 inside *SEPS1* gene) in North-East Algeria's population that belongs to an Arab-Berber (Chaouis) ethnic group.

## Methods

### Subjects

Our case-control study included 226 subjects (100 patients with HT and 126 healthy controls), from the North-East region of Algeria (Batna, Khenchela and Tébessa) that belongs to an Arab-Berber (Chaouis) ethnic group. Participants were recruited over a period of 2 years (from January 2016 to February 2018). All HT patients were recruited from three private endocrinology clinics: El Balsem el Chafy (Khenchela), endocrinology clinic of Dr. Mallem and Dr. Heddar (Batna) and the blood samples were taken at the different laboratories: Saad Laoud laboratory, Nezar laboratory and Ibn Sina laboratory in Batna province, and laboratory in El Balsem el Chafy clinic in Khenchela province. The diagnosis of HT was established by specialist of nuclear medicine based on clinical examination, ultrasound and characteristic parameters following the European thyroid association (ETA) recommendations and guidelines for the Management of Subclinical Hypothyroidism 25. The inclusion criteria for patients with HT patients were: characteristic diffuse thyroid disease on ultrasonography and increased TSH (>4.7 mIU/L), and elevated TPOAB levels (>34 IU/mL). All the patients included in the present study were hypothyroid (high TSH level) and were treated with levothyroxine therapy (Merck KGaA, Frankfurter Str. 250, Darmstadt, Germany). We excluded patients with a personal history of any other autoimmune disease. Clinical and demographic characteristics of HT patients are presented in Table 1.

**Table 1:** Clinical and demographic characteristics of HT patients and healthy controls

Characteristics	HT (N=100)	Control (N=126)	*P*-value
Gender, n (%) Men Women	4 (4%) 96 (96%)	45 (35.7%) 81 (64.3%)	<0.001^a^
Age (years)*	39.5 (32.5 – 47.75)	33 (28 – 40)	<0.001^b^
BMI (kg/m^2^) *	27.26 (24 – 30.19)	24.73 (23.04 – 26.68)	<0.001^b^
**TPOAb level (UI/mL)** *	411.5 (245.60 – 600)	11.1 (7.87 –15.00)	<0.001^b^
**TSH (µIU/mL)** **	20.68±2.54	2.44±0.08	<0.001^c^

**HT:** Hashimoto's thyroiditis; **BMI:** body mass index; **TPOAb:** Autoantibodies against thyroid peroxidase; **TSH:** thyroid-stimulating hormone; Qualitative data are presented in percentages (%)

* Quantitative data that did not follow the normal distribution is presented by a median with an interquartile range (25th – 75th percentile)

** Quantitative data that followed the normal distribution is presented by the mean ± standard deviation

a χ2-test

b Mann–Whitney-U test

c Student's t test.

Control participants were healthy individuals without any autoimmune or thyroid diseases. They were randomly selected at the blood transfusion department of the Center Hospitalization University (CHU) of Batna. All control participants had TPOAb negative (5–34 UI/mL), TSH levels between 0.27–4.7 µIU/ml, and fT4 levels between 12–22 pmol/l. Clinical and demographic characteristics of the controls are presented in Table 1.

### Ethical approval

Written informed consent was obtained from all study participants. The study was in accordance with the Declaration of Helsinki and approved by the local Scientific Ethical Committees of the Center Hospitalization University of Batna under the number 524.

### Blood samples, DNA extraction and Genotyping

Peripheral venous blood from patients with HT and control participants was collected in EDTA tubes and stored at -80° at the Laboratory of Biotechnology of Bioactive Molecules and Cellular Pathophysiology (LBMBPC), university of Batna 2, Algeria. Genomic DNA extraction was performed from the whole blood using the QIAamp DNA Blood Mini kit (Qiagen, Hilden, Germany) according to the manufacturer's instructions. Genotyping of the two SNPs (rs7537605 and rs2866512) was performed by quantitative real-time PCR with ABI PRISM 7500 Sequence Detection System (Applied Biosystems, Foster City, USA) and pre-developed TaqMan SNP genotyping assays.

Genomic DNA extraction and genotyping of the two SNPs were performed at the Laboratory of the Department of Medical Biology, School of Medicine University of Split, Croatia.

The DNA concentration was measured using the Nanodrop ND-1000 spectrophotometer (ND-1000, Thermo Fisher Scientific, Waltham, MA, USA), and the final concentration of the isolated DNA varied between (11.56 ng/µl) and (58.66 ng/µl) for 200 µl of whole blood.

### Statistical analysis

We tested the Hardy-Weinberg equilibrium of tested SNPs in our population using an exact test 26. The allelic association of each SNP with HT was tested by a binary logistic regression model adjusted for age, and the genotype frequencies between HT patients and controls were calculated using a chi-square test or Fisher's exact test. Differences between groups were determined by the odds ratio (OR) and 95% confidence interval (95% CI). In addition to the basic allelic test, we performed a full association test provided by statistical package PLINK 27 to evaluate possible models of association with disease. These included dominant, recessive and genotypic models. The dominant model analyses dominant homozygote (AA) and heterozygote (Aa) versus (aa), whereas recessive model analyses AA versus (Aa, aa). Finally, we carried out genotypic model analyses (AA versus Aa versus aa). The A/a stand for two alleles of SNP. We assessed the allelic association of these two SNPs with TPOAb levels in the group of HT patients only using a linear regression model adjusted for age with previously performed logarithmic transformation to ensure normal distribution.

We used Bonferroni's corrected P-value of 0.025 (0.05 corrected for 2 SNPs) for considering significant associations. All statistical tests were performed by PLINK software version 1.07 27 and IBM SPSS Statistics for Windows, version 20.0. (Armonk, NY: IBM Corp).

## Results

The two SNPs (rs7537605 inside VAV3 gene and rs28665122 inside SEPS1 gene) analysed in our study were in Hardy-Weinberg Equilibrium (P> 0.05/2) in both groups of HT patients and healthy controls (Table 2).

All frequencies, together with results of allelic and genotype association analysis of rs7537605 inside the VAV3 gene and rs28665122 inside SEPS1 gene between healthy controls and HT patients, are presented in Table 3 and Table 4 respectively.

**Table 2:** Characteristics of the 2 SNPs in HT patients and healthy controls

SNPs	Chr	Gene	Position	A1	A2	MAFHT^a^	MAF controls^b^	*P* (HWE) HT^c^	*P* (HWE) control^d^
**rs7537605**	1	*VAV3*	107800465	A	G	0.225	0.234	0.774	0.619
**rs28665122**	15	*SEPS1*	101277522	T	C	0.255	0.234	0.433	0.458

**SNP**: single nucleotide polymorphism; **Chr**: chromosome; **A1**: minor allele; **A2**: major allele

a **MAF**: minor allele frequency of HT patient group

b **MAF**: minor allele frequency of healthy control group

c ***P*** -value of the Hardy – Weinberg Equilibrium test for HT patient group

d ***P*** -value of the Hardy – Weinberg Equilibrium test for healthy control group.

**Table 3:** Results of allelic and genotype association analysis of rs7537605 in HT patients and healthy controls

		Controls N (%)	HT N (%)	OR (95% CI)	*P*-value
**Allele**	G A	193 (76.59) 59 (23.41)	155 (77.50) 45 (22.50)	*Referent allele* 0.969 (0.606–1.549)	0.896
**Genotypes**	GG AA AG	75 (59.52) 8 (6.35) 43 (34.13)	59(59) 4(4) 37(37)	*Referent genotype* 1.573 (0.452–5.479) 0.914 (0.524–1.595)	0.477 0.752
**AA+AG vs.GG** **AA vs. AG+GG**		51/75 8/118	41/59 4/96	— 0.614 (0.179–2.102)	1.0 0.555

**G**: major allele; **A:** minor allele; **HT:** Hashimoto's thyroiditis; **OR:** odds ratio; **CI:** confidence interval; *P* significant when ≤ (0.05/2) for the allelic association; ≤ (0.05/3) for genotype association.

**Table 4:** Results of allelic and genotype association analysis of rs28665122 in HT patients and healthy controls

		Controls N (%)	HT N (%)	OR (95% CI)	*P*-value
**Allele**	C T	193(76.59) 59 (23.41)	149(74.5) 51(25.5)	*Referent allele* 0.958 (0.599–1.532)	0.859
**Genotypes**	CC TT TC	72 (57.14) 5 (3.97) 49 (38.89)	57(57.30) 8 (8) 35(35)	*Referent genotype* 0.495 (0.154–1.594) 1.108 (0.636–1.932)	0.230 0.717
**TT+TC vs.CC** **TT vs. TC+CC**		54/72 5/121	43/57 8/92	— 2.652 (0.774–9.076)	1.0 0.138

**C:** major allele; **T:** minor allele; **HT:** Hashimoto's thyroiditis; **OR:** odds ratio; **CI:** confidence interval; *P* significant when ≤ (0.05/2) for the allelic association, ≤ (0.05/3) for genotype association.

Table 3 shows the results of the analysis of the allelic and genotype association of rs7537605 inside VAV3 gene in HT patients and the healthy controls. The frequency of the minor allele A was slightly lower in HT patients than in the healthy control group (22.5% vs. 23.41% respectively). That result, in addition, confirmed that the differences in allele frequencies between HT patients and healthy controls were not statistically significant [OR: 0.969, CI (95%): 0.606–1.549, P=0.896].

The frequency of the AA genotype was relatively higher in the control group (6.35%) than in the group of HT patients (4%). Therefore, with the wild-type GG homozygote as a reference model, we did not observe statistically significant risk for the disease occurrence and the genotypes AA and AG (P=0.477, P=0.752, respectively). Furthermore, there was no significant difference in dominant model (AA+AG vs.GG) P=1, or recessive model (AA vs. AG+GG) P=0.555 between HT patient and control group (Table 3).

Genotype analysis of rs28665122 inside SEPS1 gene, shows that the majority of HT patients (57.30%) and healthy controls (57.14%) had the CC genotype, and we also observed a similar percentage in HT patients and healthy controls with C allele (74.5%, 76.59%) respectively (Table 4).

The present study found no association of any of the genotypes and allele of rs28665122 inside SEPS1 gene with HT in a cohort of HT patients and controls. Interestingly, even in the dominant (TT + TC) and recessive (TC + CC) models, there was no significant difference between HT patients and healthy controls (P=1.0) and (P=0.138) respectively (Table 4).

Furthermore, the results of our study indicate the absence of the association of the minor alleles of two tested SNPs (rs7537605, rs28665122) with TPOAb levels in the group of HT patients only (Table 5).

**Table 5:** Results of association analysis of tested SNPs with TPOAb levels

SNP	Minor allele	TPOAb	
		
		*β* (SE)	*P*-value
rs7537605	A	6.592 (46.95)	0.888
rs28665122	T	3.769 (42.71)	0.929

*β*: linear regression slope coefficient; SE: standard error.

## Discussion

We performed a replication study of two large genetic studies from Santos et al.^21^ and Oryoji et al.^23^, to investigate the association of rs28665122 inside *SEPS1* gene and rs7537605 inside *VAV3* gene with HT in a different ethnic group that those previously reported.

Rs7537605 polymorphism inside *VAV3* gene, located on chromosome 1, probably plays an important role in thyroid function, where its mutation causes HT and hypothyroidism in Caucasian and East Asian populations but not in the tested Algerian population.

The *VAV3* gene is part of the VAV gene family, which proteins (Vav1, Vav2, Vav3) are guanine nucleotide exchange factors (GEF) for the GTPases of the Rho / Rac / Cdc42 family 28. Movilla and Bustelo 29 indicated that the *VAV3* gene is widely expressed in the thyroid gland and according to Fujikawa et al.^30^ and Dumont et al.^31^. This gene has an important role in the immune system where it contributes to the development of T and B lymphocytes cells.

The study by Oryoji et al.^23^ reported an association between the *VAV3* gene and HT, which is contradicts results of our study. In fact, Oryoji et al.^23^ conducted a comparative GWAS to determine the genetic basis that distinguishes HT from GD for a better understanding of the differences between these two genetically related thyroid autoimmune diseases. In addition, they performed the genotype association analysis of the rs7537605 polymorphism for 1363 healthy controls, 444 HT patients, and 546 GD patients from Japan and the results revealed that this polymorphism is significantly associated with HT (P = 1.24 × 10–5, OR: 1.60, 95% CI 1.30–1.97) but not with GD (P = 0.50, OR: 0.94, 95% CI 0.79–1.13), suggesting that this SNPs specifically affects susceptibility to HT 23. Another study from Eriksson et al.^32^, that included 3736 patients with hypothyroidism and 35546 controls from Europe, showed the association of the rs4915077 polymorphism inside the *VAV3* gene with hypothyroidism. Truly, tested polymorphism in the study of Eriksson et al.^32^ is in a strong linkage disequilibrium with the rs7537605 (r2 = 0.88) targeted by our study. Teumer et al.^33^ found that the rs17020122 polymorphism located in the intronic region of the VAV3 gene and located 14 kb from rs7537605 (D ‘= 0.993, r2 = 0.3182), was strongly associated with the increased levels of TSH which is a key marker for hypothyroidism. Furthermore, the analysis performed by Pickrell et al.^34^ indicated the presence of another polymorphism onside the *VAV3* gene (rs17020055) located about 6kb from rs7537605 (D'= 0.834, r2= 0.285) which was also strongly associated with hypothyroidism. Similar results like ours were reported for rs28665122 inside *SEPS1* gene by study of Xiao et al.35 which performed the genotyping of 359 HT patients and 938 healthy controls, where they revealed no significant association between the minor allele of the tested SNP and HT [P = 0.148, OR (95% CI): 1.325 (0.904–1.943)]. Genetic studies performed on AITD have shown the existence of a common genetic background between HT and GD 36 - 38, and almost 74% of polymorphisms linked to GD have the same effect on HT 24. Results of the above-mentioned study performed by Xiao et al.^35^ on 701 GD patients are in line with ours. They did not identify any significant genotype association [P = 0.432, OR (95% CI): 1.134 (0.828–1.554)] and the minor allele association [P = 0.297, OR (95% CI): 1.168 (0.872–1.566)] of the rs28665122 polymorphism with this disease.

In contrast, the relationship between the *SEPS1* gene (rs28665122) and HT has been found in European and Chinese populations. A case-control study that included 997 subjects (481 AITD and 516 controls) showed that the minor allele A frequency is more common in HT subjects than in controls (46.2% vs 28.1%, P = 5.0× 107; OR (95% CI): 2.22 (1.67–2.97) 21. Another related study performed in the Chinese population (Han ethnic group) on 1013 HT patients and 2998 healthy controls revealed that the minor allele A of the rs28665122 was positively correlated with this thyroid disorder [P = 0.000518, OR (95% CI): 1.28 (1.11–1.47)] and that result was replicated also in another study 39.

Interestingly, SEPS1 gene encodes a transmembrane selenoprotein that have a potential role in inflammation control. A thyroid contains a high concentration of selenium which reflects the importance of selenium for hormone synthesis and thyroid metabolism. Selenium is integrated into the molecular structure of the enzyme glutathione peroxidase (GPx) which plays an indispensable role in protecting the gland from oxidative damage. Selenium can regulate serum levels of TSH, FT3, FT4, TgAb and TPOAb which can eventually lead to an immunomodulatory effect^40^,^41^.

Finally, we can conclude that the results of the effect of the rs7537605 inside *VAV3* gene and the rs28665122 inside *SEPS1* gene in our population suggested that the rs7537605 polymorphism may not be the etiologic mutation, if we compare them with different effects that were reported in the other populations, especially if we consider the multifactorial nature of HT. In fact, ethnicity could be the explanation for the inconsistency of such associations, especially because it is known that ethnicity is an HT modifier and that incidence of HT is highest in Caucasians^42^. Also, rs75376505 is in linkage disequilibrium with other polymorphisms that have shown associations with autoimmune hypothyroidism in other studies. The differences in the nutritional status of selenium may be a contributing factor to this result because results were compared with other studies performed in a different geographical location with a different seleniumtatus. There are other genetic SNPs that may play a role in the aetiology of this disease in different populations.

There are several limitations in our study that should be mentioned: the sample size that included 226 participants is probably not large enough to detect the significant associations as well. Further studies and evaluations with a relatively larger sample size are necessary for further replication of our results in the Algerian population. Another limitation includes lack of nutritional data and the assessment of selenium status among our participants. This is the first genetic study to investigate the association of two SNPs rs7537605 and rs28665122 inside *VAV3* and *SEPS* genes with HT in North-East Algeria's population that belongs to an Arab-Berber (Chaouis) ethnic group. Our results suggest that the two SNPs may not be involved in the pathogeneses of HT, but further research is required to confirm this hypothesis.
